# Samuel Hahnemann*Delivered before the Alumni Association of Hahnemann Medical College, of Chicago, in March, 1892; before the faculty and students of Hering Medical College of Chicago, in March, 1893, and before the Missouri Institute of Homœopathy, at Kansas City, in April, 1893.

**Published:** 1894-10

**Authors:** H. P. Holmes

**Affiliations:** Omaha, Neb.


### SAMUEL HAHNEMANN.*

H. P. Holmes, M. D., Omaha, Neb.

In the district of Chursachsen, one of the most beautiful and picturesque regions of Germany, and in the village of Meissen, lived an industrious, God-fearing German, by name Christian Gottfried Hahnemann, by trade a painter on porcelain. Here, with his wife, whose maiden name was Johanna Chrintiane Spiess, who was esteemed a most exemplary woman, he worked for the maintenance of his family, and ever strived to serve God and his fellow-men. "He had sound and original ideas on what should be called good and worthy in men, and his doctrine was to do and to be without striving after mere appearances." "Wherever an opportunity offered for doing good, there he was sure to be, helping heart and hand, and often unnoticed." He was the author of a small work on water-color painting.

On the 10th of April, 1755, the hearts of this German couple were gladdened by Heaven's priceless gift of their first-born—a boy—in whom the interest of this lecture centres—a boy on whose head rested greater possibilities than on that of any child born to mortal parents, and who was destined to sway the thoughts of the world in the common interests of humanity for centuries to come. This child, born without the stars heralding his coming, was christened by his parents—Samuel Christian Frederick Hahnemann.

The boy was the idol of his father's heart, and he inherited all those noble qualities which had made his parent the respected citizen of Meissen. Born in a region of strikingly beautiful scenery and with a natural love for the beauties of Nature's handiwork, Samuel became at an early age an ardent admirer of the works of the Creator, and this love for his Almighty Father constantly increased as he grew older.

---

*Delivered before the Alumni Association of Hahnemann Medical College, of Chicago, in March, 1892; before the faculty and students of Hering Medical College of Chicago, in March, 1893, and before the Missouri Institute of Homœopathy, at Kansas City, in April, 1893.

The father and mother easily taught Samuel to read and write. At the age of eight years he was placed in the *stadt* (or common) school, and at the age of sixteen he entered the *furstenschule*. He became the favorite pupil of the principal, Magister Müller, and he made such progress in his studies that at the age of twelve years he was commissioned by the principal to teach to others the rudiments of the Greek language. Hahnemann, in speaking of his teacher, Magister Müller, says: "He loved me as his own child, and left me to enjoy liberties in the method of my study for which I still feel grateful to him, and which visibly influenced my subsequent labors. During the private lessons which he gave to his boarders and to myself he would listen kindly to remonstrances on my part bearing on the interpretation of classic authors, and often accepted my translation in preference to his own. I had admission to him at all hours of the day, and I was often, in many respects, openly given preference to others. It is remarkable, however, that I had the affection of all my fellow-students nevertheless. All this in consideration receives greater importance when we remember that it happened at a *furstenschule* in Saxony. While here I took pains to read less, and to thoroughly grasp in my mind before reading further."

With a family which rapidly increased to ten children, the father was not able to give them the education he desired, and he often took Samuel away from the *stadtschule* for years together, and stoutly opposed his son's taking a college course or even continuing very long at the *furstenschule*. It is related that at one time he forbade Samuel using the family lamp for his studies after the rest of the family had retired. But the boy, nothing daunted, fashioned a lamp out of a lump of clay, successfully coaxed his mother for oil, and in secret did the studying his boyish heart craved. When the father refused to furnish means for the farther education of his son, Magister Müller interceded for the boy, and, as an inducement, refused to accept any farther fees, and thus Samuel continued his attendance upon the *furstenschule*—an institution similar to our high schools in this country—until twenty years of age. Hahnemann's last essay on leaving this school was on a subject of his own choosing, "The Wonderful Construction of the Human Hand."

On Easter, 1775, Hahnemann left home for Leipzig to enter upon the study of medicine, with about $15.00 in money, the last he ever received from his father. Through the kindness of Bergrat Poerner, who understood the circumstances of his poverty, the lecture fees were all remitted, and for two years Hahnemann studied medicine in Leipzig, choosing such lectures as seemed most useful to him. During this period he was much of the time at work in various ways to earn the means necessary for his support while pursuing his studies. He was fortunate in finding an opportunity to instruct a young and rich Prussian in the French and English languages, and he also made many translations from the English for publication. Hahnemann worked so industriously that his labors enabled him to support himself, pay all his incidental expenses, and to save quite a sum besides. In order to both earn his living and attend all his classes, he slept only every other night.

His thirst for a knowledge of practical medicine induced him to leave Leipzig for Vienna, where there were better advantages, as there was no hospital or infirmary in Leipzig.

About the time of his going to Vienna, he was swindled out of his hard-earned savings by the treachery of a supposed friend. In mentioning this fact in his short autobiography, Hahnemann says: "Repentance calls for reconciliation, and I therefore refrain from giving name and circumstances." This left him with only some $29.00, which was all the money he had, and he was forced to leave Vienna after a stay of only nine months. He was fortunate again in here making the acquaintance of Liebartz von Quarin, physician-in-ordinary to the Emperor, to whom Hahnemann says he was "indebted for that which made him a physician. I had his affection and I might say his friendship. I was the only one of my time who was given the privilege of accompanying him in his calls on private patients. He loved and taught me as if I had been the only one of his pupils in Vienna, and even more, yet he never could expect any recompense from me." When Hahnemann's poverty compelled him to leave Vienna, von Quarin was influential in procuring for him a position with Baron Bruckenthal, of Hermanstadt, as family physician and librarian. Here he remained one and three-fourths years, mastering several languages and doing much additional scientific studying. He arranged the Baron's immense library and his imcomparable collection of antique coins. With the means he had earned at Hermanstadt, he departed for Erlangen, in order to complete his medical studies, and here, on the 10th of August, 1799, he defended his thesis and received the degree of *Doctor of Medicine.*

After graduating Hahnemann says: "A Swiss could not be drawn more irresistibly toward his lofty Alps than a Chursachsen to the land of his birth." He therefore chose the mining village of Hettstadt, in Mansfeld territory, and there located in order to begin the practice of medicine in the year 1780. The town, however, proved too small to satisfy him, and at the end of nine months he left for Dessau, where we find him in the spring of 1781. Here he seemed to do well and improved his time by devoting especial attention to chemistry and to the working of the mines in that region; but at the end of the year he again moved and located in Gommern, near Magdeburg. Here he did not do well as, he said, there had never been a physician there and the people did not seem to think they needed one. However, while in this place, December 1st, 1783, he married Henrietta Leopoldine Kuchlerine, step-daughter of the apothecary Hæseler of Dessau. Among the wedding gifts presented to the bride was one from Hahnemann's father. It was an ivory fan painted by himself. "Upon this the master is depicted as treating his first (lady) patient, sitting at her bed and giving her a spoonful of medicine. The other side shows the same lady recovered and sitting in her family circle. It is a charming little *genre* picture with its fine painting and its faithful portrait likeness." Of the character of Hahnemann's wife, there are two impressions. One that she led her husband a Socrates and Xantippe sort of a life. But among those who knew her best, Mrs. Hahnemann's memory is cherished for her noble qualities, and she was respected as a faithful wife, a fond mother, and a woman who was devoted to the interests of her husband and children. A careful review of all the circumstances of her wedded life is sufficient to convince any one that the good woman was greatly maligned. She willingly followed her husband in all his various wanderings and frequent removals. When poverty threatened she was steadfast, and when the world turned against her husband she remained at his side the devoted wife faithful to the last. In times of great need she parted with her jewels, bedding, and personal wardrobe for the maintenance of her loved ones, and was the cloud by day and the pillar of fire by night that guided and encouraged the great man on his difficult and disheartening way. She died March 31st, 1830, in Cöthen, where Hahnemann was living practically in exile.

From Gommern Hahnemann removed to Dresden in the year 1785. In this city he found still better facilities for study and here made the intimate acquaintance of the City Physician Wagner, whom he refers to as "a model of incorruptible integrity." They became great friends, and, on the failing health of Wagner, Hahnemann, with the approval of the magistrate, succeeded to all his hospitals, and speaks of it as "a wide field for one charitably inclined." He seemed to enjoy his life here very much, for he says: "Four years in Dresden and vicinity thus passed for me more rapidly than they would to the unexpected heir of great wealth. In the midst of my growing family and to be nearer the source of the sciences I came to Leipzig about the time of Michælmas, 1789, where I calmly awaited the act of Providence to decide what fortune would be apportioned to each day of my life, the length of which lies in His hands."

It seems, however, that the real motive which led Hahnemann to Leipzig had a deeper significance than "to be nearer the centres of knowledge." Already he had sounded the depths of allopathic lore, and found it a shallow pool compared to the unfathomable gulf of erudition and mystery it was supposed to be. While in Gommern he had written a work containing an account of his experience as a practitioner in Transylvania. It gives a sombre view of the medical practice in general, and in reference to his own success in particular Hahnemann said that most of his patients would have done better had he left them. He became more and more discouraged over the results of his medical practice, for he observed that the treatment in the majority of cases was worse than useless. So that, when he removed to Leipzig, it was virtually the abandonment of his work as a practitioner of medicine. He says: "It was painful for me to grope in the dark, guided only by our books, in the treatment of the sick—to prescribe according to this or that fanciful view of the nature of diseases, substances that owe to mere opinion their place in the materia medica. I had conscientious scruples about treating unknown morbid states in my suffering fellow-creatures with these unknown medicines, which, being powerful substances, may, if they were not exactly suitable [and how could any physician know whether they were suitable or not, seeing that their peculiar special actions were not yet elucidated?] easily change life into death, or produce new affections or chronic ailments which are often much more difficult to remove than the original diseases. To become in this way a murderer or aggravator of the sufferings of my brethren of mankind was to me a fearful thought. So fearful and distressing was it that shortly after my marriage I abandoned practice, and scarcely treated any one for fear of doing him harm, and, as you know, occupied myself chiefly with chemistry and literary labors."

Shortly after settling in Leipzig, he published his work on Syphilis, and in it introduced to the profession the preparation still known by the name of "Mercurious Solubilis Hahnemanni." He gave a careful and accurate description of its mode of manufacture, administration, etc.

We come now to the year 1790—the year more fraught with interest to humanity than any yet recorded in the archives of scientific progress. It was the year of freedom coming to the benighted condition of medical knowledge. The year of emancipation in which the medical light, so long hidden in mystery and charlatanism, should shine forth to the world, and the shackles of blind custom and traditional medicine should be struck from its practitioner, and he should be left free to use the mind God had given him, and to pursue a better way in the treatment of his suffering fellow-men.

Great consequences sometimes grow out of trivial circumstances. The falling of an apple led to the discovery of the law of gravitation; the singing of a teakettle was the first hint of the power hidden in steam; taxation without representation led to the freedom of America; the shadow on the moon led Galileo to believe the earth was round; on the refusal of a request that his wages be raised a half-dollar a month, Fernando Magellan changed employers and so became the first to circumnavigate the earth; on the shake of a peasant's head rested the fate of Bonaparte and the French at Waterloo; and the obscurity in the description of a single remedy (Cinchona) led Hahnemann to found the grandest law in therapeutical medicine: *Similia Similibus Curantur.*

In the year 1790 Hahnemann was working at his translations for the purpose of earning his daily bread. Cullen's *Materia Medica* had been placed in his hands to be rendered from English into German. Happy day for medical science! Happy day for the millions of sick and suffering humanity in the ages to come! We homœopaths should erect a monument to the memory of Cullen, for he it was who was the unconscious author of our origin. As one after another of the remedies in this work came under Hahnemann's critical observation, he was more and more struck with the uncertainty and obscurity of the knowledge of medicine and medicinal action. He longed to raise the profession from the bogs and mists of uncertainty and to place it upon a sure and rational foundation. When he came to Cinchona, he felt more than ever impressed with the empty phantoms and mania for worthless hypotheses in the art of healing of that day. He determined to test the action of the drug in the only sure and reliable manner, viz.: upon himself. He took a large dose of the famous remedy and observed the symptoms. Strange to relate, on that same day he suffered a veritable attack of ague or intermittent fever. He experimented upon others with the same result. Then came the question: Should not the power of Cinchona which produced intermittent fever in a healthy person be the same as that which cured the disease? He experimented again and again with different remedies and with similar results. The query settled into a conviction, and the conviction into a law which he thus formulated: "Diseases are healed in the safest, easiest (least painful and most convenient) manner, as well as permanently, through such medicines as produce in the healthy body effects as near as possible resembling those of the disease. Or, one must endeavor to heal diseased conditions with such remedies as produce in the perfectly healthy body diseased conditions most similar in character." This theory forms the centre and basis of a school which has convulsed the whole science of medicine, the essence of which is the law *Similia Similibus Curantur*.

Hahnemann was now anxious to thoroughly test his new theory, but his poverty compelled him to labor with his pen for the support of his family. Fortune soon favored him in an offer from the reigning Duke of Saxe-Gotha to take charge of the insane asylum at Georgenthal in the Thuringian forest. As this gave him a competent income and ample leisure for study, he thankfully accepted the position. While at the head of this institution he succeeded in curing the Hanoverian Minister, Klockenbring, who had been rendered insane by a lampoon written by Kotzebue. The description of Klockenbring's insanity is a masterpiece in its way. Hahnemann is supposed to have been the first to advocate the treatment of the insane with kindness, and never allowed his patients to be punished by blows. The same year, however, Pinel unchained the maniacs in the Bicetre, and it is a disputed question as to whom should belong the honor. We know, nevertheless, that Hahnemann did it working independently, and to-day the custom is universal in all well-governed insane asylums.

He did not remain long in Georgenthal, and made several removals between 1792 and 1795; to Walschleben, Pyrmont,

Brunswick, and to Wolfenbuttle; from there to Konigslutter, where he lived for four years. All this time he was busily engaged in writing and translating, proving remedies and evolving the science of the new system. He freely published his researches and their results, deeming that his first duty always lay in giving his knowledge to his profession. Like all great reformers, however, he was greeted with a storm of abuse and opposition instead of receiving the thanks of his medical brethren. At Konigslutter he had been phenomenally successful in the treatment of scarlet fever with Belladonna, and this, with his growing popularity, aroused such a jealousy among his *confrères* that they induced the apothecaries to prefer charges against Hahnemann for dispensing his own remedies. The authorities issued an order enforcing the law against the self-dispensing of remedies, and Hahnemann, in the morning of his fame, was forced to quit the town in the year 1799. Think of this, ye practitioners of medicine who are beginning life's struggle in your profession. Samuel Hahnemann, forty years of age, a scholar and student, a man of giant intellect, a man who was master of more languages than the average physician had medical books in his library, whose *Apothekerlexicon* was the handbook on the German druggist's table, who taught the pharmacists the profession they followed; think of this man being driven from town to town because it was claimed he was not competent to dispense his own medicines!

On leaving Konigslutter, a crowd of people composed of friends and patients followed the wagon which contained Hahnemann's wife, family, and earthly possessions a long way on the road to Hamburg. Then a sad farewell was taken, the people thanking God for the benefit Hahnemann had been permitted to confer upon them. On this journey the wagon, in descending a precipitous portion of the road, was overturned. A daughter had an arm broken, and their infant son, Ernst, was so seriously injured that he died shortly afterward. Can you, my hearers, doubt of the bitterness that must have come to the heart of this great man over this loss of his darling child? Near where this accident occurred the family were detained six weeks on account of the injury of the daughter. At length they reached Hamburg, where Hahnemann could do but little. Then on to Altona; next to Mollen; then back to Saxony, where he tried to practice in Eilenberg. Persecution compelled him to move. He tried Machern, and next Dessau. During all this time he worked nightly, for the support of his family, in the translation of medical and scientific works. Poverty crowded upon him, and history says he was reduced to such straits that he was obliged to weigh out to each member of his family their portion of bread, that each one might fare justly. A little girl was taken ill, and asked that a portion of brown bread be put away for her so that she might have it when she was better.

Hahnemann prosecuted his investigations in the science of medicine in spite of poverty and persecution. He ceased working at translations, and from 1806 devoted his time to original articles. "Æsculapius in the Balance," "A Pure Materia Medica," and the "Medicine of Experience," came forth rapidly. These productions raised such a storm of opposition, and the author was so reviled that he gave up trying to educate the medical profession, and for a time wrote only for the benefit of the people. He published his subsequent articles in a literary and scientific journal.

We come now to the eventful year of 1810. Hahnemann was living in Torgau. He was fifty-five years of age, had been a doctor of medicine since 1779, and had been developing the law of Similia Similibus Curantur for twenty years. Truly, enough experience for a scientist to know what he was doing. He published his greatest work, *The Organon*, which was soon read in more languages than any book save that of Holy Writ. It ran through five editions, and was translated into English, French, Italian, Spanish, Hungarian, Polish, Russian, Danish, and Swedish. In this work for the first time was used the word *Homœopathy*.

The name of Samuel Hahnemann was now a celebrated one throughout all Europe. In 1811 he removed once more to Leipzig, and adherents flocked to him from every direction.

Anxious to teach his new theory to the academic students, he sought permission and was informed he could do so by writing and defending a dissertation and by paying the sum of fifty thalers. On the 28th day of June, 1812, Hahnemann gave his famous dissertation in Latin on the "Helleborism of the Ancients." It was a masterpiece of scholarship and scientific knowledge, and produced a great sensation in Leipzig. One of his hearers, a Dr. Huck, says: "To hear Hahnemann, the boldest investigator of nature, defend the masterpiece of his genius and diligence, was an enjoyment truly heavenly. As I rode home in a dream, I felt the solitude about me when I had to admit that I was unworthy to loosen his shoes. He covered himself with glory. He remained victor."

At the delivery of this dissertation, Frederick Hahnemann acted as respondent. We will pause in our history to recite what is known of the life and fate of this the only son of the founder of Homœopathy.

Frederick Hahnemann, born in Dresden the 30th day of November, 1786, was the pride of the family, and whose fate was so peculiar and so appealing to our sympathy as to make his life one of unusual interest to us. He was a stout, healthy little fellow as a child, and was familiarly called Fritz by his father. He had a brilliant education, spoke Latin, Greek, French, English, and Italian and was conversant with Arabian, was a fine musician and a general favorite with all who knew him. He graduated in medicine at Leipzig, in 1812, although the year before he had entered the field as an author in defending his father against Hecker's attack upon *The Organon*. He purchased a drug-store at Wolkenstein, in order to be freed from the law against the self-dispensing of drugs, and there began the practice of his profession. His fame quickly spread, and his house was thronged with patients. He lived in pompous style and went from place to place with a coach and four. He contracted a matrimonial alliance with a widow, which gave great offense to his father, and this estrangement was never quite removed. Frederick Hahnemann's professional success soon aroused a jealousy among his allopathic *confrères*, and a charge was brought against him of dispensing his own remedies. Though his cause was a good one and he could have undoubtedly won his case if he had remained for trial, he would not give his enemies the satisfaction of appearing before them, and so determined for once and all to avoid their persecutions and left home, wife, children, country, and practice to take up his life in a foreign land. He went to England and sent letters home at irregular intervals. These letters were so odd, the arrangement of the written matter on the page was so eccentric, and the mode of expression was so peculiar that his family were firmly convinced of his insanity. A letter written from London, in the spring of 1819, was so filled with this recklessness, so unusual to the careful student, that his father exclaimed in the most vehement grief: " My poor son is becoming insane !" The last line was written by Frederick from London, June 25th, 1820, and he was forever afterward lost to the knowledge of his family. The father bent under the heavy grief and several times expressed his fear that his son had died in an insane asylum. Report has it that in 1830 a traveler calling himself Frederick Hahnemann had visited the interior of Pennsylvania, and had cured many persons by means of small powders. Nothing more definite was ever known of him.

For about nine years Hahnemann remained in Leipzig, winning many laurels, practicing continually, and constantly working to perfect Homœopathy. He had many distinguished friends and illustrious patients. But envy and malice were at work against him. The apothecaries made a government complaint against him for dispensing his own medicines. In a rescript in December, 1820, the authorities admitted the justness of the apothecaries' complaint, and deprived Hahnemann of the right to dispense his own remedies. This, of course, destroyed the effectiveness of his labors, although it did not absolutely prohibit his practicing his profession. But crippled in his professional privileges, he was forced to say farewell to his native country.

Hahnemann's words regarding his new system were a veritable prophecy. He said : " Our art needs no political lever, no ribbons of worldly orders to develop it into something. Gradually it grows through the many weeds, sprouting high around it, from the unostentatious acorn into the vigorous sapling." The reigning Duke, Ferdinand, of Anhalt-Cöthen, offered him a home in his city and appointed him Hofrath, or Court Physician. Hahnemann removed to Cöthen early in the summer of 1821. He felt the restraint of his smaller field, and was so embittered against the world as, for a long time, to seldom pass the threshold of his own door. He devoted himself with even greater assiduity to perfecting his system and recording his researches. His fame followed him and his practice was very large. People from all over Europe, from the United States, and even South America came to him for treatment, and many physicians made Cöthen their Mecca to study the new school of medicine. Hahnemann was soon so crowded with business that he had to take an assistant in order to accomplish the work that was daily thrust upon him.

While in Cöthen a reunion was held which had a great significance for Hahnemann and Homœopathy. It was on the 10th of August, 1829, the fiftieth anniversary of Hahnemann's graduating in medicine. It was his Doctor's Jubilee. Friends from far and near gathered to do honor to the great man. In a beautifully decorated room he was presented with a finely written programme of the occasion, a beautiful medal engraved by Kruger, of Dresden, with the portrait of Hahnemann, the date of his birth and graduation on one side, and his homœopathic axiom, *Similia Similibus*, on the other; a large oil portrait of Hahnemann well executed by Schöppe, of Berlin; the congratulatory diploma from the faculty at Erlangen, together with many other tokens and expressions of regard. The venerable jubilant, with heartfelt emotion, gave thanks to his Almighty Father that he had been permitted to make so important a discovery. Congratulatory letters were received from the Duke and Duchess of Anhalt-Cöthen, accompanied by the present of an antique cup and a golden casket ornamented with the initials of the Duke in brilliants. At this meeting was organized the first Homœopathic medical society, the "Central Society of German Homœopathists," which was henceforth to meet annually on the 10th of August.

In 1831, when the cholera broke out in Europe, Hahnemann carefully studied the symptoms of the disease through the reports sent him, and decided upon Camphor as the indicated remedy. Reports poured in from physicians, priests, and laymen in regard to the wonderful efficacy of Camphor, and the concensus of opinions showed that hardly a death occurred where the remedy was used according to instructions. Hahnemann did not see a case of cholera during the epidemic. A finer illustration of the workings of our law of cure cannot be found. To prescribe for a malady without seeing it, to cover the picture of the disease with a remedy whose pathogenetic symptoms show it to be the simillimum, and to effectually stamp out the epidemic was a victory such as the medical world had never witnessed.

Hahnemann remained in Cöthen fourteen years, one of the busiest and most honored scientific men in all Europe. We find him at the close of 1834 an old man of seventy-nine years, active, strong, and full of the faith in his new school. His wife had been in her grave for five years. A lady patient had come from Paris to consult the great Hahnemann in regard to a serious affection of her heart and lungs. Her name was Melanie d'Hervilly Gohier, an adopted daughter of Louis Gerome Gohier, who was Minister of Justice and President of the Executive Directory of the French Republic in 1799. This lady was an enthusiast in all that interested her. She was a daring rider and swimmer, possessed all kinds of rifles, had obtained permission to hunt, had been in the academy of painting, in some way had clandestinely been admitted to the dissecting-rooms, and had taken a course of lectures on anatomy. A mutual admiration sprang up between this young Frenchwoman and the learned doctor, and it is facetiously stated that Hahnemann cured her of her lung trouble, but changed the heart affection into another one of a more chronic nature. They were married on the 28th of January, 1835.

The young wife, with her usual enthusiasm, was anxious to have her husband locate in Paris, and so fascinated the old man with her alluring representations of the fame and honor he would reap in France that he resolved to leave his fatherland and follow his wife to Paris. A farewell trip was taken to Leipzig, where Hahnemann gave a dinner to his pupils and a reception to the great man was held. Early on the morning of the first Whitsunday, 1835, they left Cöthen for Paris, and Germany never again saw the beloved founder of Homœopathy.

In Paris, Homœopathy was struggling for a foot-hold. There were a few representatives, but little was known of the new system. "*Elle est morte!*" or "*On n'en parle plus!*" were the common expressions regarding it. The coming of Hahnemann was to raise the banner on high and establish the cause on a sure foundation. By a royal decree, obtained through the kindness of M. Guizot, Hahnemann received permission to practice. One newspaper after another took up the cause and rallied to the support of Hahnemann and Homœopathy. The following year a medal in likeness of Hahnemann was cast, and a deputation waited upon him to present him with it as a token of their honor for him and to thank him for locating in their country.

Patients flocked to see the great man and to secure his services. It is said that Hahnemann valued his advice very highly, as it required ten *Louis d'or*, or about $48 to consult him. Be this as it may, he also devoted a great deal of time to treating charity cases. He occupied a large hotel and the patients came to see him in veritable processions. Helen Berkeley, an American lady who visited Hahnemann professionally, gives her experience as follows: "In 1839, Dr. Hahnemann was residing in Paris, near the garden of the Luxembourg. During the winter of that year, desiring to consult him in behalf of an invalid friend, I made him my first visit. That I might obtain an audience as early as possible, I entered the carriage which was to transport me to his residence at a quarter past nine o'clock in the morning. After about half an hour's ride, finding that the coachman stopped his horses without dismounting, I inquired if we had reached our destination; 'No, madam, it is not our turn yet. We must wait a little while. See! there is Dr. Hahnemann's house,' he replied, pointing to a palace-like mansion at some distance. This mansion was surrounded by a mossy stone wall, with an iron gate in the centre. Impatient at the delay, I leaned out of the window and beheld a long line of carriages in front of us, driving through the gate, and out again, as fast as their occupants alighted. This was vexatious; I had taken such especial pains to be early—and all to no purpose. But if there was any consolation to be found in the knowledge that others were even worse off than ourselves, I might have comforted myself by looking in the opposite direction. Behind us stretched a file of coaches, lengthening every minute, and already quite as formidable as the one in front. I had unconsciously taken my station in the midst of a procession slowly advancing to pay homage to the modern Æsculapius. I already knew something of Hahnemann's celebrity; but my opinion of his skill was marvelously fortified as I stared behind me and before me, and then at the empty carriages driving away around me.

"In about twenty minutes the carriage in which I sat wondering and waiting, during that time having moved forward a few paces every minute, at last drove briskly through the iron gate, around the spacious court, and deposited us, to my great satisfaction, at the front entrance of Hahnemann's magnificent dwelling. Three or four liveried domestics, assembled in a large hall, received the visitors as they alighted, and conducted them to the foot of the side staircase. At the head of the first flight they were received by a couple more of these bedizened gentlemen, who ushered them into an elegant saloon, sumptuously furnished, and opening into a number of less spacious apartments."

Farther on, Mrs. Berkeley states that she waited three hours in the parlors before her turn came to visit Dr. Hahnemann.

Such was the ability of our great leader in his new home. His private life was filled with those events which go to make pleasant the life of one who has won fame through his own endeavors. Birthday celebrations, visits from admiring followers and indefatigable attention to perfecting his great work, filled his time to the fullest. His earnings were said to be 200,000 francs or about $40,000 annually, and that for a man well past four-score years of age.

Hahnemann's life was now nearing its end, and he was awaiting the Master's call, content to give up the struggle and be at rest with Him in whom he had so long trusted. July 28th, 1839, he had written of himself: "*Non inutilis vixi*"—I have not lived in vain. Later on he wrote to a friend: "It is perhaps time that I quit this life, but I leave all and always in the hands of my God." On another occasion he wrote: "My conscience is clear; it bears me witness that I have ever sought the welfare of suffering humanity, that I have always done and taught what seemed to me best, and that I have never had recourse to any allopathic procedures to comply with the wishes of my patients, and to prevent them leaving me. I love my fellow creatures and the repose of my conscience too much to act in that manner. Those who follow my example will be able, as I am, on the verge of the grave, to wait with tranquillity and confidence till the time comes when they must lay down their heads in the bosom of the earth, and render up their soul to a God whose omnipotence must strike terror into the heart of the wicked."

The sun of his life set in glowing colors. Each spring-time for some years past Hahnemann had been subject to an attack of bronchial catarrh. The 15th of April, 1843, he was taken so seriously ill with his old trouble that his wife admitted no one. Several times he was reported as dead and the statements as many times disproved. This condition ran on through the rest of the months of April, May, and June with variations from better to worse. Hahnemann's mental faculties were retained to the last and he gave advice regarding the choice of remedies for his affection that showed the wonderful memory, knowledge, and judgment of the man. He was treated by his wife and Dr. Chatran. He suffered more from attacks of dyspnœa toward the end. It was during one of these attacks that his wife said to him: "Providence should really owe to you an exemption from your sufferings, because you have alleviated those of so many others, and have borne so many hardships in your laborious life." He replied: "To me? Why to me? Every one in this world works according to the talents and powers which he has received from Providence, and more or less are words used only before the judgment-seat of man, not before the throne of Providence. Providence owes me nothing, but I owe it much. Yes, everything!"

"He retained his mental faculties to the last moment, and though his voice grew more and more indistinct, his broken words showed the continued clearness of his mind and the calmness with which he saw his end approaching. At the very beginning of his illness he had told his people that this would be his last, as his frame was worn out."

Samuel Hahnemann died at five o'clock on the morning of July 2d, 1843, aged eighty-eight years two months and twenty-two days. A mistake as to this date was probably caused by a typographical error. The only two works published in the German language relative to the date of his death, *Hahnemann's Life and Works*, by Albrecht, and *History of Homœopathy*, by Ameke, give the day as June 4th. But the cuts of the monument erected to his memory in Leipzig, as well as other authorities, give it as July 2d. To settle the question, I wrote to Dr. R. E. Dudgeon, of London, who should know more of Hahnemann than any English-speaking homœopath. He replied: "I thought the best way to answer your question was to ask his grandson, Dr. L. S. Hahnemann. He tells me that the true date of his grandfather's death is July 2d, 1843, and he should know, as he was at the old man's funeral, if not exactly 'in at the death.'"

At this time, Jahr writes: "I had repeatedly resolved to call there when I received a note from Mrs. Hahnemann, asking me to call on her that day. I went immediately, and was admitted at once to Hahnemann's bed-room. But imagine my horror, instead of there finding Hahnemann, the dear, friendly old man, greeting me with his smile, I found his wife stretched out on his bed in tears, and next to her him lying cold and stiff, having passed five hours before into that life where there is no strife, no sickness, no death. Yes, dear friends, our venerable father has finished his course. Paralysis of the lungs has freed his spirit from its tired frame after an illness of six weeks."

Hahnemann had no public funeral. His remains were embalmed by Ganal, taken to the cemetery of Montmartre on the rainy morning of July 11th, 1843, and buried near the left of the entrance. Only his wife, one daughter and her son, and the servants followed the body to the grave.

One month after Hahnemann's death, the Central Society of German Homœopathists met in Dresden and resolved to erect a monument to the beloved leader. The statue of Hahnemann was executed by the sculptor Steinhauser and then cast in bronze in Rome. With appropriate ceremonies the corner-stone was laid in Leipzig and the completed monument unveiled and dedicated on the 10th of August, 1851. Hahnemann, living in Leipzig, was persecuted and driven from the city. Dead, his memory was cherished in the hearts of his followers and his fame emphasized in marble and bronze. His was a life well spent—a benefactor to suffering mankind, a physician, and a true minister of God. He lives with us to-night, and thousands of orisons ascend heavenwards to bless the memory of the greatest physician of past or modern times—Samuel Hahnemann, the sage of Cöthen, the founder of Homœopathy.

